# Transcriptome Analyses of *Procambarus clarkii* (Girard, 1852) Under Individual Exposures to CuSO_4_, Pendimethalin, and Glyphosate

**DOI:** 10.3390/toxics13090765

**Published:** 2025-09-09

**Authors:** Yao Zheng, Jiajia Li, Zhuping Liu, Ning Wang, Gangchun Xu

**Affiliations:** 1 Wuxi Fisheries College, Nanjing Agricultural University, Key Laboratory of Freshwater Fisheries and Germplasm Resources Utilization, Ministry of Agriculture and Rural Affairs, Freshwater Fisheries Research Center (FFRC), Chinese Academy of Fishery Sciences (CAFS), Wuxi 214081, China; 13357933502@163.com (J.L.); liuzp@ffrc.cn (Z.L.); 2 Xinghua Modern Agriculture Development Service Center, Taizhou 225700, China; wangning@ffrc.cn

**Keywords:** hepatopancreas, lysosome, peroxisome, oxidative phosphorylation, herbicide

## Abstract

Pesticide usage in the integrated rice–crayfish system has aroused lots of attention all over the world. Especially in China, fish farmers often use copper sulfate and pendimethalin to remove moss from aquaculture water and glyphosate to remove weeds in and around crayfish–crab mixed culture ponds. To explore the stress response mechanism of CuSO_4_, pendimethalin, and glyphosate to the hepatopancreas of *Procambarus clarkii* (Girard, 1852), seven treatment groups including control, CuSO_4_ (1 and 2 mg·L^−1^), pendimethalin (PND, 5 and 10 μg·L^−1^), and glyphosate (5 and 10 μg·L^−1^) experimental groups were set up; the transcriptome responses were detected at 4, 8, and 12 days, respectively. The irregular structure and vacuoles were shown in the hepatopancreas for 2 mg·L^−1^ CuSO_4_ and 10 μg·L^−1^ glyphosate exposures at 12 d, while narrowed hepatic sinusoids were revealed after 10 μg·L^−1^ pendimethalin exposure. The pathways of ribosome, lysosome, and peroxisome were significantly enriched for differential expression genes (DEGs); in addition, tyrosine metabolism, starch, and sucrose metabolism were enriched under the stress of the three inputs. Genes in related pathways such as glycerophospholipid metabolism, oxidative phosphorylation, and glycerolipid metabolism also changed, and the expression of genes associated with oxidative phosphorylation changed significantly under the stress of the three inputs. Oxidative stress, neurotoxicity, metabolism, and energy supply have been significantly affected by the above herbicide exposure. High concentrations and/or long-term duration exposure may result in metabolic disorders rather than eliminate toxicity through adaptability responses.

## 1. Introduction

*Procambarus clarkii* (Girard, 1852), commonly known as red swamp crayfish [1], was introduced into China in the 1930s. It has a strong survival period, adaptability to the environment, and reproductive ability. Additionally, it has become the largest economic freshwater crustacean in China because it is highly preferred by consumers. In recent years, with the advancement of intensive aquaculture, an outbreak of disease in crayfish and crab has occurred. The farmers often use CuSO_4_ (copper sulfate, CAS 7758-98-7) and pendimethalin (PND, CAS 40487-42-1) to remove moss and glyphosate (CAS 1071-83-6) to remove weeds growing in and around aquaculture water from crayfish–crab mixed culture ponds. The world’s largest consumer market for CuSO_4_ was the Asia Pacific region, followed by North America, South America, and Africa with the fastest growth rates, which can be used for controlling harmful algal blooms and off-flavors. At present, the global market demand for PND exceeds 40,000 tons, with Europe and Asia accounting for 28.5% and 27.3% of the global market share. In 2024, the total global use of glyphosate was estimated to reach 871,700 tons, with Argentina, the United States, and Brazil all using over 100,000 tons. Prolonged use of these drugs in large quantities can adversely affect the growth of crayfish. In this paper, the effects of exposure to the three inputs (CuSO_4_, PND, and glyphosate) on the transcriptomes of crayfish hepatopancreas were discussed.

The water treatment and disease control compounds commonly used in aquaculture can reduce the innate immunity and, therefore, disease resistance of crayfish [2]. Seventy-two h LC_50_ values for crayfish were 0.54 mg·L^−1^ for CuSO_4_, and the order of Cu bioaccumulation was gill, hepatopancreas (without histological changes), and muscle [3]. The total mean concentration of Cu in pond water and range in sediment was 4 μg·L^−1^ and 21.3–45.7 mg·kg^−1^ [4]. Oxidative stress in the crayfish has been induced after copper nanoparticle exposure [3], while the different histological changes between the gill and hepatopancreas and the mode of action on toxicological mechanism in the crayfish hepatopancreas have not been determined.

PND is a dinitroaniline preemergent herbicide widely used to control grasses and weeds; PND in water systems worldwide indicate a range of 100–300 ng·L^−1^, but levels have been reported as high as ~15 µg·g^−1^ in sediment, which may produce damage in the neural and reproductive systems [5]. The histopathological examination of liver tissues of treated bighead carp (*Hypophthalmichthys nobilis* (Richardson, 1845)), showed mild to moderate congestion, necrosis of hepatocytes, and atrophy of hepatocytes under 0.75 mg·L^−1^ PND exposure [6]. The results of sub-lethal toxicity on PND for early stages of fish embryos and larvae showed a high prevalence of spinal curvature, tail malformations, pericardial edema, and yolk sac edema at 4 dpf at 25 μM [7]. After 0.5 mg·L^−1^ PND exposure, musculoskeletal development is affected, leading to delayed and reduced ossification of the vertebral centra in the developing vertebral column and disruption of muscle morphology with increased AChE activity [8], which can be detected in fish muscle, like in the common carp, crucian carp, eel, and Chinese muddy loach [9]. With PND as the primary component, the herbicide’s product name is fluchloralin, which can lead to mitochondrial dysfunction in zebrafish [10]. However, after the PND mixture’s pesticide exposure, heat stress co-exposure significantly impacted natural swimming patterns [11] and apoptotic cells appeared in the kidney [12] and gills of goldfish [13]. PND is rated as third after glyphosate and paraquat, and its tolerance for red swamp crayfish in the USA has been set at 0.05 mg·kg^−1^, while the tolerance in Chinese integrated rice–crayfish systems has not been determined.

Glyphosate has been found in the surface water (61.4 µg·L^−1^ with a 100% detection rate), sediment (46.5 ng·g^−1^ with a 100% detection rate), and organisms (6.55 ng·g^−1^·dw^−1^ with a 57% detection rate) of the crayfish in ponds around Lake Honghu [14]. Except for genotoxic potential in spermatozoa by 90 μg·L^−1^ [15], concentrations of 5~20 mg·L^−1^ of glyphosate induced considerable neurotoxic and immunotoxic effects in red swamp crayfish for 96 h [16], and another 1.2~10.8 mg·L^−1^ glyphosate after 72 h of exposure showed that the antioxidant capacity, ammonia-nitrogen regulation, and energy supply of the organism was enhanced [17], while 0.1~10 µg·L^−1^ glyphosate alerted neurotoxic and oxidative impacts for 14 d as a longer exposure duration [18]. The capacity of tolerance for long-term exposure duration and molecular mechanism in red swamp crayfish needs to be investigated, especially in the integrated rice–crayfish system with amounts of usage of pesticides.

The total products for red swamp crayfish are 2.89 million tons, and the total area for rice fishing farming is 44.9 million mu in 2024 in China, while the usage for herbicide is 0.28 million tons in 2023, which may directly cause harm the largest lobster production and humans who consume it. The current study aims to test the toxicological mechanism of CuSO_4_, PND, and glyphosate using red swamp crayfish as an animal model.

## 2. Materials and Methods

### 2.1. Chemicals Stock Preparation, Animals and Sample Collection

CuSO_4_ (pure, highly concentrated, 99%) was purchased from Sinopharm Group (Beijing, China), pendimethalin (PND, emulsion form with 40% purity) was purchased from Wuxi Zhongshui Fishery Medicine Co., Ltd. (Wuxi, China), and glyphosate (emulsion form with 30% purity) was purchased from Bydis Australia Limited (Canberra, Australia). Red swamp crayfish *Procambarus clarkia* (Girard, 1852) (*n* = 252, 81.69 ± 3.65 mm, 21.31 ± 2.07 g) was taken from the Xianghu grain planting family farm in Xinghua City, and after 7 days of temporary rearing in the laboratory (maintained in a plastic tank with a diameter of 10 m and height of 1.5 m), the robust and healthy crayfish were selected and placed in tanks (63 cm × 43 cm × 45 cm) [1], each containing fifteen L of aerated dechlorinated tap water [1], and the water temperature was controlled at (18.5 ± 2.3) °C during temporary rearing and the experiment. The pH was 7.5 ± 0.6, the dissolved oxygen was 8.6 ± 2.9, the daily light-to-dark ratio was 12 h:12 h, and nitrogen and phosphorus contents met the fishery water quality standard.

The 72 h LC_50_ value for CuSO_4_ in crayfish was 0.54 mg·L^−1^, and CuSO_4_ could be detected in water and sediment samples as 0.14 mg·L^−1^ and >10 mg·kg^−1^ [19]. The usages of 0.18–3.2 mg·L^−1^ in winter flounder and 0.5–2.5 mg·L^−1^ in tilapia for 21 d of exposure have been selected [20]. Additionally, 0.1–0.75 mg·L^−1^ PND and 0.1 µg·L^−1^–10.8 mg·L^−1^ glyphosate (with 0.06 µg·L^−1^ detection in water) have been selected for exposure. The concentrations of the three inputs have been selected based on the above reported data for earlier warning. Seven groups (12 ind. per each triplicate tank) were divided in triplicates for each treatment, which were named as the control group (A1), CuSO_4_ (1 and 2 mg·L^−1^, named as group B1 and B2), pendimethalolin (PND, 5 and 10 μg·L^−1^, named as group C1 and C2), and glyphosate (5 and 10 μg·L^−1^, named as group D1 and D2). During the experiment, continuous oxygenation was maintained, and the breeding environment temperature was adjusted using an air conditioner. In compliance with FFRC-CAFS rules, animal welfare was given top priority (LAECFFRC-2021-04-08). The daily feeding amount was based on the technical specification for crayfish, which was 4% of the body weight of the experimental crayfish. Feeding and body weight changes were adjusted when necessary. The exposure time duration has been selected based on the reported data from 24 h to 42 d. After the feeding test, aseptic sampling was carried out at the exposure periods of 4, 8, and 12 days (for transcriptomics in groups, added 1, 2, and 3 to A11, A12, and A13 of 4, 8, and 12 days for the four treatment groups from A to D). During sampling, twenty-seven crayfish (*n* = 12, 36 ind. in total) for treatments were collected from each glass tank to meet the sampling amount of hepatopancreas tissue for histopathological (*n* = 6, H&E), transcriptomics (*n* = 3), and qPCR verification (*n* = 3, the primers are revealed in Table A1) to demonstrate the effects between long- and short-exposure durations. Crayfish were starved for 24 h before sampling and biological measurements were then performed following MS-222 anesthesia.

### 2.2. Histopathological Alterations

Following a 24 h fixation in a 4% formaldehyde solution, each crayfish hepatopancreas (*n* = 6) sample was used for H&E staining in the manner previously mentioned [21] using a rotary microtome (Leica RM2235, Leica Microsystems, Wetzlar, Germany). Simply, the samples group underwent conventional washing, gradient dehydration, transparency, wax dipping, and embedding using 5 μm thick slices. Following standard dewaxing, gradient dehydration, H&E staining, drying, and neutral gum sealing, the slices were inspected under an Olympus CHC binocular light microscope (Olympus Corporation, Tokyo, Japan).

### 2.3. Transcriptomics and qPCR Verification

The transcriptome sequencing and analysis were conducted by OE biotech Co., Ltd. (Shanghai, China), Nanjing Baokairan Biotechnology Co., Ltd. (Nanjing, China) according to the manufacturer’s instructions as previously described (American life technology company, Seattle, WA, USA) [21]. Total RNA was extracted (TRIzol^®^ Reagent, Invitrogen, Carlsbad, CA, USA) and genomic DNA was removed using DNase I (TaKara, San Jose, CA, USA). RNA integrity was evaluated (RNA Nano6000 detection kit, Agilent Bioanalyzer 2100 system, Agilent Technologies, Santa Clara, CA, USA), the RNA concentration was determined (American life technology company, USA), and samples with RNA integrity number values larger than 7 were selected. We took the transcriptome spliced by Trinity as the reference sequence, and estimated the gene expression level of each sample through RNA-seq by Expectation–Maximization (RSEM): aligned clean data to the assembled reference sequence, obtained the read count number of each gene according to the alignment results, standardized the read count data with the trimmed mean of *M*-values (TMM), and then conducted the analysis with DEGs. With regard to data analysis, raw data removal, gene function annotation, and gene expression estimation, differently expressed gene analysis (DEGs), gene ontology (GO), and the Kyoto encyclopedia of genes and genomes (KEGG) enrichment analysis were followed by the reported references [21]. The screening threshold was *q*-value = 1. To identify the affected genes under the three input exposures among the comparisons (i.e., A11 vs. B11, A11 vs. B21, etc.), we screened significant differentially expressed genes (DEGs) in the KEGG pathways associated with ABC transporters, drug metabolism-cytochrome P450, and oxidative phosphorylation using qPCR as previously reported [21]. This study selected *β*-actin as the reference gene and computed changes in mRNA levels (*n* = 3).

### 2.4. Data Statistical Analysis

All experimental data were analyzed using SPSS software (version 26.0), and its value is expressed as mean ± standard deviation, while after log_2_ treatment, data without a homogeneous distribution were analyzed. The data of different groups were statistically analyzed by one-way ANOVA and the Tukey–Kramer test. *p* < 0.05 was used to indicate significant difference.

## 3. Results

### 3.1. Histopathological Changes

**Figure 1:**
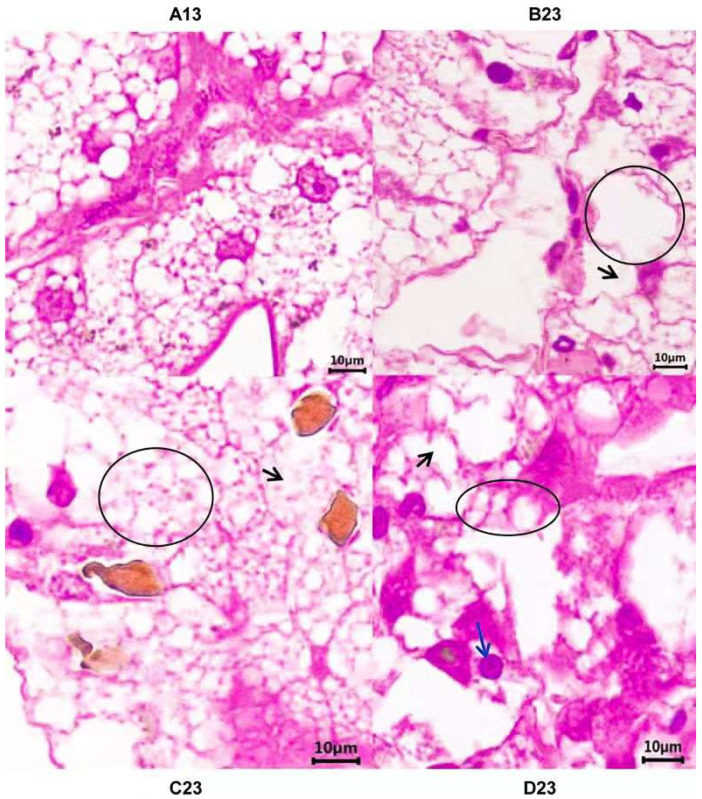
The histopathological changes caused by 2 mg·L^−1^ CuSO_4_ (B23), PND (C23), and glyphosate (D23) (*n* = 6). A13, controls; in B23, B, 2, and 3 stand for the CuSO_4_ group, the higher concentration like 2 mg·L^−1^, and 12 days, respectively. The black circle and arrow showed vacuoles. In C23, C, 2, and 3 stand for the PND group, the higher concentration like 10 μg·L^−1^, and 12 days, respectively. In D23, D, 2, and 3 stand for the glyphosate group, the higher concentration like 10 μg·L^−1^, and 12 days, respectively. The black circle showed narrowed hepatic sinuses without clear cell outlines with the black arrow in C23, and the blue arrow showed the narrowed bile canaliculus in D23. The black arrow showed vacuoles.

For 2 mg·L^−1^ CuSO_4_ and 10 μg·L^−1^ glyphosate exposure at 12 d, irregular structure and vacuoles showed in the hepatopancreas (Figure 1), while after 10 μg·L^−1^ PND, narrowed hepatic sinusoids were revealed, and compressed bile canaliculi was also present for glyphosate exposure. Within the quantitative assessment of Bernet [22], the ratio of vacuoles significantly increased in 2 mg·L^−1^ CuSO_4_ (24.3 ± 2.5%) and 10 μg·L^−1^ glyphosate (31.4 ± 1.6%) exposure groups, when compared with the controls (6.4 ± 0.5%). The area proportion of vacuoles in PND and the controls were 29.7 ± 3.6% and 16.8 ± 2.7%. The number of compressed bile canaliculi in glyphosate and the controls were 2.4 ± 0.2 × 10^−2^ ind.·(cm^2^)^−1^, 0.0 ± 0.0 × 10^−2^ ind.·(cm^2^)^−1^.

### 3.2. Transcriptomics Data

**Figure 2:**
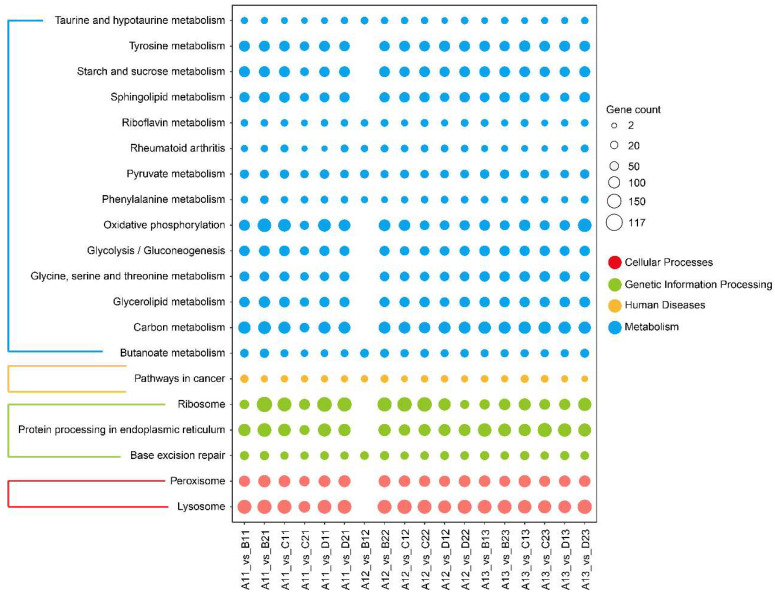
The KEGG pathway enrichment via transcriptomics among the different treatment comparisons (*n* = 3). X- and Y-axes show different comparisons and different enriched KEGG pathways; the different colors show the same categories for their enriched KEGG pathways. The size of the circle represents the number of enriched DEGs.

In this study, 2372, 2484, and 2387 DEGs have been found in CuSO_4_, PND, and glyphosate exposures, respectively (Table 1). The KEGG results revealed ribosome, lysosome, peroxisome, tyrosine metabolism, starch and sucrose metabolism, glycolysis/gluconeogenesis, oxidative phosphorylation, and glycerolipid metabolism-related pathways, of which peroxisome, ribosome and lysosome were the most significant pathways (Figure 2).

**Figure 3:**
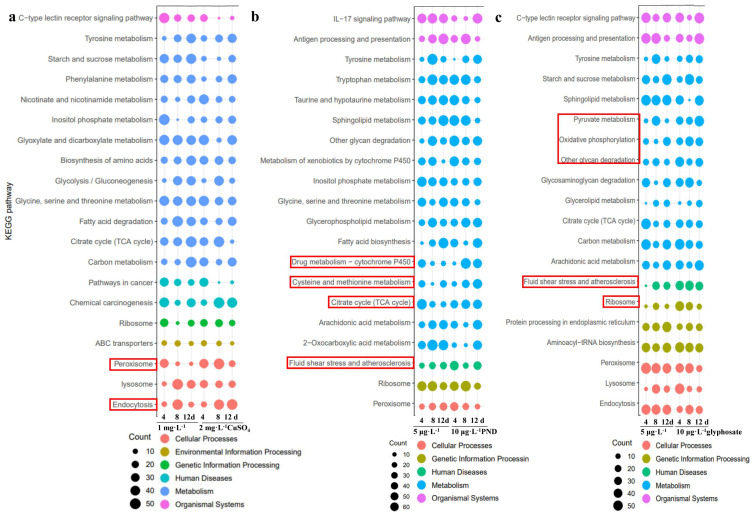
The transcriptomic effect caused by CuSO_4_ (**a**), PND (**b**) and glyphosate (**c**) (*n* = 3). X and Y-axes show different comparisons and different enriched KEGG pathways; the different colors show the same categories for their enriched KEGG pathways. The size of the circle represents the number of enriched DEGs; the red boxes show the enriched comparison groups and KEGG pathways.

**Figure 4:**
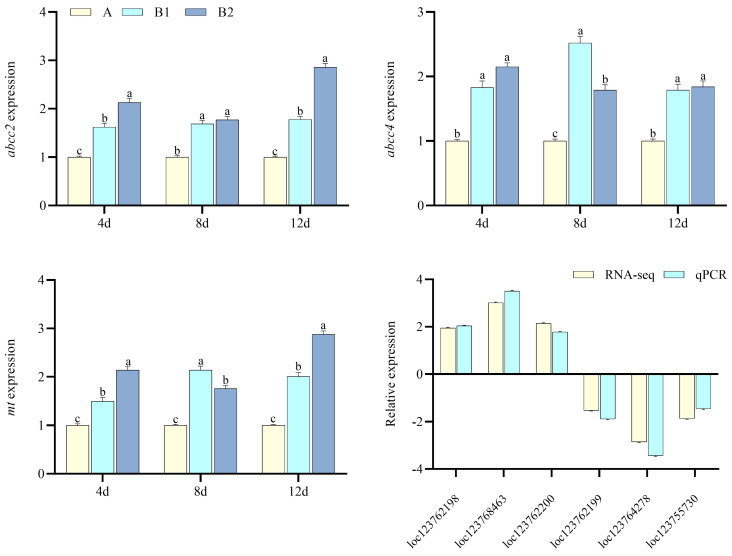
Genes verification for CuSO_4_ exposure (*n* = 3, *p* < 0.05 stands for the significance level). A, B1, and B2 stand for the control, and the 1 and 2 mg·L^−1^ CuSO_4_ groups. *abcc2*, ATP−binding cassette subfamily C member 2, *abcc4*, ATP−binding cassette subfamily C member 4, and *mt*, metallothionein. *loc123762198* (DNA−directed RNA polymerases I, II, and III subunit RPABC2 pseudogene), *loc123768463* (ATP−binding cassette subfamily A member 3), *loc123762200* (DNA−directed RNA polymerases I, II, and III subunit RPABC2 pseudogene), *loc123762199* (DNA−directed RNA polymerases I, II, and III subunit RPABC2 pseudogene), *loc123764278* (zinc finger C3HC-type protein 1-like), and *loc123755730* (ATP−binding cassette subfamily E member 1 pix) used for qPCR verification when compared with RNA-seq.

For CuSO_4_ exposure, the pathways of ABC transporters, peroxisome, and endocytosis were significantly enriched (Figure 3a). DEGs of ABC transporters and *mt* (metallothionein) were significantly increased; *abcc2* and *abcc4* significantly increased (Figure 4, *p* < 0.05); *abcc2*, *abcc4*, and *mt* were significantly higher at 12 d than at 4 and 8 d, which were significantly higher than those in controls (*p* < 0.05). Among DEGs in the pathways of ABC transporters, *loc123762198*, *loc123768463*, and *loc123762200* significantly increased, while *loc123762199*, *loc123764278*, and *loc123755730* significantly decreased.

**Figure 5:**
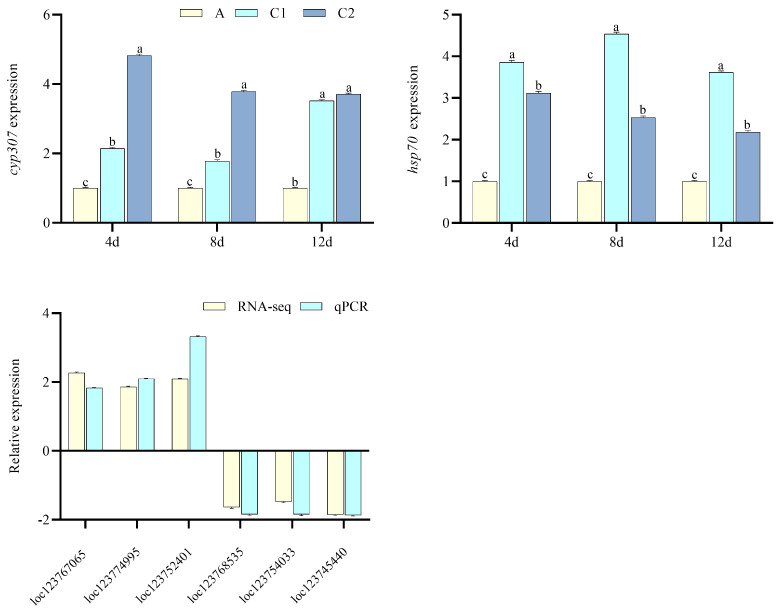
Gene verification for PND exposure (*n* = 3, *p* < 0.05 stands for the significance level). A, C1, and C2 stand for the control and the 5 and 10 μg·L^−1^ PND groups. *cyp307*, cytochrome P450 307a1−like, *hsp70*, heat shock 70 kDa protein. *loc123767065* (cytochrome P450 2L1), *loc123774995* (glutathione S-transferase theta−1), *loc123752401* (probable cytochrome P450 49a1), *loc123768535* (Cytochrome P450 4c3), *loc123754033* (UDP−glucosyltransferase 2), and *loc123745440* (glucose−fructose oxidoreductase domain-containing protein 1) used for qPCR verification when compared with RNA-seq.

For PND exposure, drug metabolism-cytochrome P450, cysteine and methionine metabolism, citrate cycle (TCA cycle), fluid shear stress, and atherosclerosis were significantly enriched (Figure 3b). *cyp307* and *hsp70* were significantly increased. Among DEGs in the pathways of drug metabolism-cytochrome P450 (Figure 5), *loc123767065*, *loc123774995*, and *loc123752401* significantly increased, which may relate with pendimethalin degradation, while *loc123768535*, *loc123754033*, and *loc123745440* significantly decreased.

**Figure 6:**
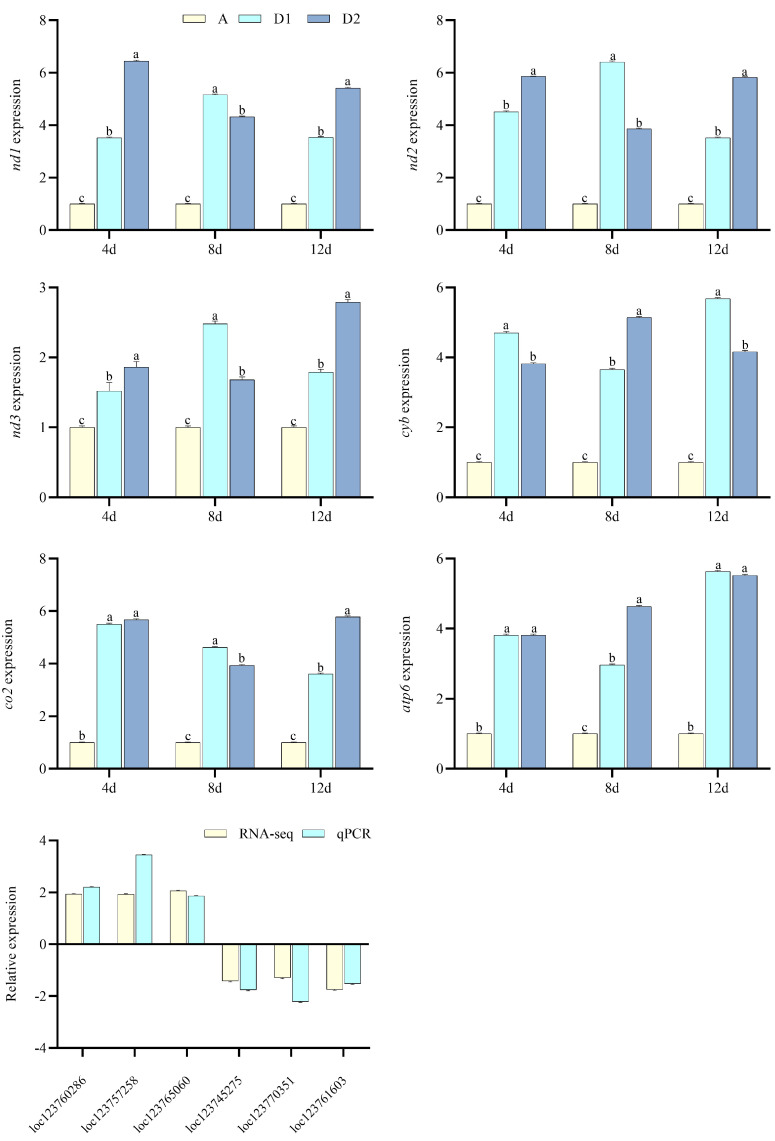
Genes associated with oxidative phosphorylation verification for glyphosate exposure (*n* = 3, *p* < 0.05 stands for the significance level). A, C1, and C2 stand for the control and the 5 and 10 μg·L^−1^ glyphosate groups. *nd1*, NADH dehydrogenase subunit 1, *nd2*, NADH dehydrogenase subunit 2, *nd3*, NADH dehydrogenase subunit 3, *cyb*, cytochrome−b, *co2*, cytochrome c oxidase subunit II, *atp6*, ATP synthase F0 subunit 6, *loc123760286* (Cytochrome c oxidase assembly factor 10), *loc123757258* (uncharacterized LOC123757258), *loc123765060* (V−type proton ATPase 16 kDa proteolipid subunit c), *loc123745275* (cytochrome b−c1 complex subunit 7), *loc123770351* (ATP synthase subunit alpha blw, mitochondrial), and *loc123761603* (NADH dehydrogenase [ubiquinone] 1 beta subcomplex subunit 2, mitochondrial) used for qPCR verification when compared with RNA−seq.

For glyphosate exposure, pyruvate metabolism, oxidative phosphorylation, other glycan degradation, fluid shear stress and atherosclerosis, and ribosome were significantly enriched (Figure 3c). Among DEGs in the pathways of oxidative phosphorylation (Figure 6), *nd1*, *nd2*, *nd3*, *cyb*, *co2*, *atp6*, *loc123760286*, *loc123757258*, and *loc123765060* significantly increased, while *loc123745275*, *loc123770351*, and *loc123761603* significantly decreased.

## 4. Discussion

Rice-based integrated farming systems with great models have attracted a lot of concern in China, which may correspond with more pesticide use for removing field weeds. The water quality and crayfish growth can be enhanced in the rice–crayfish model, which may be attributed to the diversity and structure of microbiomes and reduced opportunistic pathogens [23]. Interestingly, pesticide use decreased by 17% when compared with typical rice monoculture cultivation [24]. Considering CuSO_4_, PND for removing moss, and glyphosate for removing weeds in the rice–crayfish model, the study planned to know the harmful hepatic transcriptome response. Pathways of ABC transporters (100 μg·L^−1^ copper hydroxide nano pesticide in zebrafish) [25], drug metabolism-cytochrome P450 (in red swamp crayfish by 1.02 mg·L^−1^ cyhalofop-butyl and 10.4 mg·L^−1^ pyribenzoxim) [26], and oxidative phosphorylation (100 μg·L^−1^ in zebrafish) [27] were mainly affected by CuSO_4_, PND, and glyphosate, respectively, for 12 days. The concentrations of the three inputs in this study were larger than the actual environmental conditions for earlier warning (Table 2), and results from this study showed that higher concentrations of the three inputs took the harmful effect on histological and transcriptional changes in red swamp crayfish, which hinted that the government must pay more attention to pesticide monitoring and the assessment of the quality and safety of aquatic products in China and especially focus on its implication in the integrated rice–crayfish system. When compared with the standardized toxicity testing zebrafish, crayfish were much more tolerant, could be used as the testing alternative based on this study and others [1,3,15,16,17,18,26].

*mt* significantly increased in zebrafish [25] and red swamp crayfish in the present study. *abcc2* and *abcc4* significantly increased in red swamp crayfish with renal function found in zebrafish [30], which was similar to the current study. The drug metabolism-cytochrome P450 pathway was significantly affected by pesticide exposure, like cyhalofop-butyl [31] and glufosinate-ammonium [32] in zebrafish. *hsp70* significantly increased in fish (like the common carp) when exposed to pyrethroid insecticide (0.15 μg·L^−1^ esfenvalerate) [33], while it decreased in tilapia of our study under 0.2~16 mg·L^−1^ glyphosate [29]. In zebrafish, following 100 μg·L^−1^ glyphosate exposure, stress responses and metabolic processes, like the oxidative phosphorylation pathway [27], were significantly enriched, which was similar to this study. The respiratory chain relative genes (*nd1*, *nd2*, *nd3*, *cyb*, *co2*) significantly increased after Aflatoxin B1 [34] exposure and can be affected by temperature [35] and glyphosate in this study. The current study confirmed that for red swamp crayfish, oxidative stress, neurotoxicity, metabolism, and energy supply have been significantly affected by the above herbicide exposure.

Crustaceans, like freshwater prawn *Macrobrachium borellii* (Caridea: Palaemonidae), showed a dose-dependent manner after 0.006–0.2 μg·L^−1^ cypermethrin or 0.5–1.7 spirotetramat [36]. From the data of this study, the affected pathways were significantly enriched by long-term duration exposure for PND and glyphosate when compared with short-time exposures (Figure 3), but the ribosome pathway showed the reverse tendency by CuSO_4_ exposure with a high enrichment score at 4 d. CuSO_4_ was used to control harmful algal blooms and remove moss (together with PND) in the crayfish–crab mixed culture ponds, resulting in decreased dissolved oxygen, which significantly enhanced the ABC transporter pathway, similar to the previous study in zebrafish [25]. High concentrations of CuSO_4_ alerted harmful effects, while lesser CuSO_4_ increased the body weight of fish animals. In China, PND and glyphosate have low toxicities for fish in lower concentrations. PND and glyphosate significantly affected the gene expressions in the pathway of drug metabolism-cytochrome P450 and oxidative phosphorylation in the current study and resulted in irreversible histological changes at a higher concentration or for long-term exposure duration (Figure 1). The current study showed high concentrations and/or long-term duration exposures of the three inputs resulted in metabolic disorders with irreversible organic impairment after adaptation response with antioxidative stress and mitochondrial gene expression changes.

The limits of PND in water and sediment were 0.1~0.25 ng·L^−1^ and 0.01 ng·g^−1^, with its half-lives of 0.51–5.64 d [37,38]. Our team’s previous studies focusing on prometryn (2 mg·L^−1^) showed that a swollen lumen, oxidative stress, immunity, inflammation, and detoxification were affected for 20 d [39], which could alleviate the intestinal toxicology via the Nrf2-Keap1 and MAPK pathway [40]. In the co-existing status with pesticides, heavy metals, and other environmental pollutants, the restoration technique includes phytoremediation and anaerobic microorganisms [41]. A study showed that 125 g·kg^−1^ diet *Azolla pinnata* can protect PND’s toxicology through mitigating oxidative damage [28], which has been demonstrated in our previous study using mint for methomyl removal [42]. S-rlusulfinam and R-flusulfinam were found to preferentially accumulate in sediment, water and the overall system [43]. For pesticide manufacturers, it is urgent to develop variative pesticides with low ecological toxicities [44], which can be used in the integrated rice–fish farming system, and be healthy to humans with lower residue [45].

Even though histological impairment was found, the most significant pathways of peroxisome, ribosome, and lysosome were enriched following the three herbicide exposures; the flaw of this study is that it does not clearly identify the modes of action and the difference for each input in red swamp crayfish, like performing further studies on exposure time duration, sample size, the exposure concentrations, the sampled tissues, and the environmental factors, which may affect its histological and transcriptional changes. The bioaccumulation and residue for each input are also worthwhile to conduct in different concentrations and time spans (within and without their half-life), different crayfish culture models (integrated rice–crayfish system, crayfish–crab mixed culture, etc.), different pesticide degradation conditions, etc. Because neonicotinoid pesticides (like thiamethoxam and thiacloprid) were still detected in the rice paddy ecosystem with the ecological agriculture method [46], after that, the three pesticides impaired crayfish, raising questions about the compatibility between pest control and healthy fish production in an integrated rice cultivation and mud crayfish farming system; further research is required to ascertain the impact of current pest management practices and determine the reasonable concentration of pesticide use and the interval between pesticide withdrawal periods.

## 5. Conclusions

The rice-based integrated farming system uses much more herbicide for removing field moss and weeds, which may pose threat to the living activity of red swamp crayfish, or even to human health through the food chain. The effects of exposure to the three inputs (CuSO_4_, PND, and glyphosate) on the transcriptomes of crayfish hepatopancreas were examined. Histological slices revealed the irregular structure and vacuoles, narrowed hepatic sinusoids, and compressed bile canaliculi. The pathways of peroxisome, ribosome, and lysosome were significantly enriched for the three inputs’ exposures, while different special pathways were significantly affected among the three herbicides. For red swamp crayfish, its oxidative status, neurotoxicity, metabolism, and energy supply have been significantly affected by the above herbicide exposure. The flaw of this study may at least include not finding the reason for histological and transcriptional difference among the three inputs and its mode of actions. Further research is required to ascertain the concentration and frequency limits, expiration dates, degradation pathways, residues in crayfish, and harm to humans of pesticides used under different modes in actual production. More variative pesticides with low ecological toxicities need to be developed based on the amount usage of herbicides in the integrated rice–fish farming system of China.

## Figures and Tables

**Table 1:** Differential gene expression analysis among groups.

Comparisons	Groups	DEGs—Total	DEGs—Up	DEGs—Down
CuSO_4_ vs. control	A11_vs_B11	2664	1153	1511
	A12_vs_B12	2464	1161	1303
	A13_vs_B13	2512	1422	1090
	A11_vs_B21	2585	1579	1006
	A12_vs_B22	2561	1268	1293
	A13_vs_B23	2372	1540	832
PND vs. control	A11_vs_C11	2105	1233	872
	A12_vs_C12	2583	1257	1326
	A13_vs_C13	2711	1443	1268
	A11_vs_C21	1837	1086	751
	A12_vs_C22	2562	1237	1325
	A13_vs_C23	2484	1426	1058
glyphosate vs. control	A11_vs_D11	2362	956	1406
	A12_vs_D12	2194	945	1249
	A13_vs_D13	2527	1424	1103
	A11_vs_D21	2011	926	1085
	A12_vs_D22	2538	1134	1404
	A13_vs_D23	2387	1589	798

Note: A11, A12, and A13 are control groups at 4, 8, and 12 d, respectively. B stands for the CuSO_4_ group, the first number “1” in B11, B12, and B13 stands for 1 mg·L^−1^; the first number “2” in B21, B22, and B23 stands for 2 mg·L^−1^. The second number “1, 2, 3” stands for 4, 8, and 12 days, respectively. C stands for the PND group, C11/C12/C13 stand for 5 μg·L^−1^ PND exposure for 4, 8, and 12 days, respectively, while C21/C22/C23 stand for 10 μg·L^−1^ PND exposure for 4, 8, and 12 days, respectively. D stands for the glyphosate group, D11/D12/D13 stand for 5 μg·L^−1^ glyphosate exposure for 4, 8, and 12 days, respectively, while D21/D22/D23 stand for 10 μg·L^−1^ glyphosate exposure for 4, 8, and 12 days, respectively.

**Table 2:** Different comparison analyses for crayfish in this study when compared with others.

Inputs	Other References	Crayfish in This Study
CuSO_4_	[3], red swamp crayfish, antioxidative enzymes decreased after exposure to copper nanoparticles for 48 h but without histological changes	irregular structure and vacuoles, pathways of ABC transporters, peroxisome, and endocytosis enriched
	[25], 100 μg·L^−1^ copper hydroxide nano pesticide in zebrafish, ABC transporters pathway enriched	
PND	[6], congestion, necrosis of hepatocytes, and atrophy of bighead carp hepatocytes under 0.75 mg·L^−1^ PND exposure	irregular structure and vacuoles; pathways of drug metabolism-cytochrome P450, cysteine and methionine metabolism, citrate cycle, fluid shear stress, and atherosclerosis enriched
	[8], delayed and reduced ossification of the vertebral centra, increased AchE in zebrafish by 0.5 mg·L^−1^ PND exposure	
	[11,12,13], co-exposure with high temperature, natural swimming patterns affected, apoptotic cells in the kidney and gill of goldfish found	
	[28], oxidative damage found in tilapia under 0.5 and 1 mg·L^−1^ PND exposure	
glyphosate	[15], genotoxic potential in spermatozoa of crayfish by 90 μg·L^−1^ glyphosate exposure	narrowed hepatic sinuses, narrowed bile canaliculus, *hsp70* increased, pathways of pyruvate metabolism, oxidative phosphorylation, other glycan degradation, fluid shear stress and atherosclerosis, and ribosome enriched
	[16], neurotoxic and immunotoxic effects after 5~20 mg·L^−1^ glyphosate exposure in crayfish for 96 h	
	[17], antioxidant response, ammonia-nitrogen regulation, and energy supply of the organism enhanced in crayfish after 1.2~10.8 mg·L^−1^ glyphosate for 72 h	
	[18], 0.1~10 µg·L^−1^ glyphosate alerted neurotoxic and oxidative impacts in crayfish for 14 d	
	[27], 100 μg·L^−1^ in zebrafish, oxidative phosphorylation pathway enriched	
	[29], *hsp70* increased in tilapia under 0.2~16 mg·L^−1^ glyphosate exposure for 28 d	

## Data Availability

Additional data supporting the findings of this study are available from the corresponding author upon reasonable request.

## Appendix A

**Table A1:** The qPCR primers used in the present study.

Gene Names	Primers	Product (bp)
*β-actin*	F: GACAAGTACAGTTGTGCGCC R: TGGCCCATACCAACCATCAC	194
*abcc2*	F: CCACCTTCGCTGTGTTTGTG R: GCACTGGCTCTGTGTCTCTT	282
*abcc4*	F: CAATGAGCCAATCGCTGCTC R: TCGGCTCCATATCGCTTGTC	216
*mt*	F: AGGAAGAGGTGAACGCCTTG R: CAGGGTGTCGCTGGATTCTT	259
*loc123762198*	F: GGGAGAAGACCTTGGTGCAA R: GTGCTCTCGTGCCCAATACT	250
*loc123768463*	F: GCTGCTAGACGAGCCATCAT R: GGTCATGGCCATCTTGGGAA	269
*loc123762200*	F: AATCCGGCTGAAACGTCGAT R: GCCGTTTTCCACGTTTTTGTG	210
*loc123762199*	F: GCAAACAGGAACGCGAGATG R: AGTGCTCTCGTGCCCAATAC	235
*loc123764278*	F: ACCAGGCAACAGTCAACACA R: ACACGGCTACAGAATGCCTC	235
*loc123755730*	F: AATCCACTGTGCGCCAACTA R: AGAGAGTCAAAGCCACTCGC	162
*cyp307*	F: TCCCAGGACATCCGATCCTT R: CGTATCTTCCTGCCGACCTC	251
*hsp70*	F: TGGCCATTCGACGTCATCAA R: AGATGGTTCCAGCGTCCTTG	226
*loc123767065*	F: AGAGGAAGGACGAGCCTGAT R: GAGCTTATCGTCGAGGGTGG	212
*loc123774995*	F: AAATTTGGCGGCAGTGTTCC R: TCAGTCGTCTGCACCTCAAC	167
*loc123752401*	F: AGGATTAAACTGGTGGCGGG R: ATCCTCTGTCCCTCTTCGCT	258
*loc123768535*	F: TGTGACTTACCGCCTTCACC R: GCCTCGTGACACTCTCAACA	224
*loc123754033*	F: CTACGCATCATGGCAACGTG R: CAAGAAGGGAACTGCGACCT	245
*loc123745440*	F: GAAGCGGCAGATTGAAGCAG R: CATGCCGTGAACGCGATTAG	202
*nd1*	F: TCGGGTAGGAGACGTAGCAA R: GCAGTCACCAGAGTTGACGA	237
*nd2*	F: TTTTCTGCTTGGTTGCCAGC R: AAATTCGCCCCTAGACCTGC	194
*nd3*	F: ATCGGGTAGGAGACGTAGCA R: AGCAGTCACCAGAGTTGACG	239
*cyb*	F: GCTCCTGTGGTAGAGGTTGG R: AGCCCATGAAACCCAGTAGC	299
*co2*	F: TGATTTGGGGCTTGAGTGGG R: TGAAAAGGAGCTGCGCCTAA	227
*atp6*	F: AGGCCGCTGCTTGATATTGA R: GGTAATCGGGCCCTTCCTTT	295
*loc123760286*	F: TGGTGGTAACAGCAGTAGCG R: GGCATGTAAAGGGGTCACCA	203
*loc123757258*	F: TGCGTTCCAAATCTCTGGCT R: ATAAGGTTGACTGGGGCTGC	191
*loc123765060*	F: GGCTGAACGTCAACCTCAGA R: CCAGCCATGACAACAGGGAT	280
*loc123745275*	F: TTCTCGCCCAGCTCAACAAT R: GGCTGCAGATAACGTCCCTT	262
*loc123770351*	F: TGGCAAGACTGCGATTGCTA R: GGAAGAATTCCCCCATGGCA	254
*loc123761603*	F: CCGTCGTCCACACTATCACC R: CTGTGCGGTAAACCCATCCT	186
